# Assessing climatic conditions and biotic interactions shaping the success of *Cystoseira foeniculacea* early‐life stages

**DOI:** 10.1111/jpy.13516

**Published:** 2024-10-23

**Authors:** Alejandro Bernal‐Ibáñez, Eva Cacabelos, Raul Triay‐Portella, Patrício Ramalhosa, Ignacio Gestoso

**Affiliations:** ^1^ MARE – Marine and Environmental Sciences Centre/ARNET – Aquatic Research Network, Agência Regional Para o Desenvolvimento da Investigação Tecnologia e Inovação (ARDITI) Funchal Madeira Portugal; ^2^ Faculty of Life Sciences University of Madeira Funchal Portugal; ^3^ Hydrosphere‐Environmental Laboratory for the Study of Aquatic Ecosystems Vigo Spain; ^4^ Centro Oceanográfico de Vigo (COV‐IEO), CSIC Vigo Spain; ^5^ Grupo en Biodiversidad y Conservación, IU‐ECOAQUA Universidad de Las Palmas de Gran Canaria Las Palmas Canary Islands Spain; ^6^ Department of Biology, Faculty of Marine and Environmental Sciences & Marine Research Institute (INMAR) Universidad de Cádiz (UCA) Puerto Real Cádiz Spain; ^7^ Smithsonian Environmental Research Center (SERC) Edgewater Maryland USA

**Keywords:** climate change, grazing, marine forests, marine heatwaves, thermotolerance

## Abstract

Early‐life stages of canopy‐forming macroalgae are critical for the maintenance of natural populations and the success of restoration actions. Unfortunately, the abiotic conditions and biotic interactions shaping the success of these stages have received less attention than the interactions shaping the success of adults. Here, we combined field and mesocosm experiments to explore the effects of temperature, herbivory, and canopy presence on the development of early‐life stages of the brown seaweed *Cystoseira foeniculacea*. We assessed these effects by examining changes in recruit density and size. After recruiting zygotes under laboratory conditions, we conducted one laboratory and three field experiments. In the first field experiment, the density of recruits decreased over time in all rockpools and was negatively affected by rising temperatures and turf cover. Additionally, a marine heatwave (MHW; 11 days >25°C) was recorded in the donor pools, producing strong decay in the density of transplanted recruits and a significant reduction of the mature canopy. The second field experiment tested the survival of recruits based on their positioning within the canopy. We observed a higher density of recruits when placed at the edge or outside the canopy compared to recruits placed under the canopy. In the third field experiment, an herbivory‐exclusion experiment, we show how density of recruits decreased in less than 48 h in noncaged treatments. In the laboratory, we conducted a thermotolerance experiment under controlled conditions, exposing the recruits to 19, 22, 25, 28, and 31°C for 7 weeks to assess thermal impacts on their survival and growth. Temperatures above the 25°C threshold reduced the density and size of the recruits. This study sheds light on the performance of the early‐life stages of a *Cystoseira* spp. in Macaronesia, showing a low survival ratio against the current pressures even in the context of the potential refuge provided by the intertidal rockpools.

**Abbreviations**
ANOVA: analysis of variance
GAM: generalized additive model
LM: linear model
MHW: marine heatwave
PAR: photosynthetically active radiation
RD: relative recruit density
*SE*
: standard error
SST: sea surface temperature

## INTRODUCTION

Species belonging to *Cystoseira* sensu lato (Fucales, Phaeophyceae) have been described as indicators of good environmental status (Piazzi et al., 2018; Verdura et al., 2023). The intricate systems created by mature canopies of *Cystoseira* s.l. forests serve as vital habitats, providing shelter and sustenance to numerous associated species (Cheminée et al., 2013; Piazzi et al., 2018). However, the populations of these species are in decline in the Mediterranean Sea and the Webbnesia archipelagos of Madeira and Canary Islands (NE Atlantic Ocean; Bernal‐Ibáñez, Cacabelos, et al., 2021; Martín García et al., 2022; Valdazo et al., 2017; Verdura et al., 2023). Consequently, these foundational species are being replaced by less complex organisms, leading to significant shifts in benthic communities (Bernal‐Ibáñez, Cacabelos, et al., 2021; Casado‐Amezúa et al., 2019; Pessarrodona et al., 2021). These shifts have led to the prevalence of less productive and structured assemblages, including, for example, sea urchin barrens, turf‐forming algae, and other opportunistic species (Kletou et al., 2018; Pessarrodona et al., 2021). The complete loss or fragmentation of these forests poses a severe threat to biodiversity and ecosystem services, directly impacting coastal economic activities (Eger et al., 2021; Verdura et al., 2023).

The decline of *Cystoseira* s.l. populations has been linked to multiple anthropogenic pressures, such as habitat fragmentation, urbanization, and overfishing of sea‐urchin's predator species (Airoldi, 2003; Martín García et al., 2022; Thibaut et al., 2005); it has also been linked to climate change (Bernal‐Ibáñez et al., 2022; Verdura et al., 2021). Information concerning the natural recovery of impacted *Cystoseira* populations is limited (Iveša et al., 2022; Medrano et al., 2020; Orlando‐Bonaca & Rotter, 2018) because once losses have occurred, recovery relies on nearby populations and is hindered by the limited dispersal of zygotes and the low connectivity between populations (Assis et al., 2024; Capdevila et al., 2015; Verdura et al., 2018). Furthermore, in the current climate change scenario, thermal anomalies and warming are shown to alter the reproductive phenology, viability of recruits, and the performance of *Cystoseira* s.l. mature individuals (Bevilacqua et al., 2019; Capdevila et al., 2018; Celis‐Plá et al., 2017; Verdura et al., 2021). As documented, the Mediterranean Sea (Verdura et al., 2021) and the NE Atlantic Ocean (Bernal‐Ibáñez, Cacabelos, et al., 2021) are undergoing accelerated warming compared to other regions worldwide. This is evident in the increasing frequency, intensity, and duration of thermal anomalies (Bernal‐Ibáñez et al., 2022; Oliver et al., 2019), which are restructuring coastal rocky bottoms. Examining the response of *Cystoseira* s.l. species to temperature may provide helpful insights into the potential resilience of populations to future climate change scenarios.

Multiple studies have explored the population dynamics and factors impacting the survival of mature individuals of *Cystoseira* s.l. populations (Benedetti‐Cecchi & Cinelli, 1992; Capdevila et al., 2015, 2016; Irving et al., 2009; Thibaut et al., 2016). However, the mechanisms driving their successful recruitment remain poorly understood. Previous studies have addressed the potential role of the presence of adults in the settlement and survival of juveniles (Capdevila et al., 2015). This knowledge is crucial for understanding how natural and human‐induced disturbances may lead to the fragmentation of *Cystoseira* s.l. populations or even their complete collapse. Understanding the mechanisms influencing *Cystoseira* early‐stage development is essential for the effective management of these crucial species, enabling the optimization of restoration efforts (Cebrian et al., 2021; Verdura et al., 2018).

Herbivores are essential regulators of benthic macroalgal communities in rocky reef systems (Alves et al., 2003; Ling et al., 2010; Vergés et al., 2014). Although the dynamics of macroalgal forests depend on abiotic variables, such as light or temperature, the role of herbivores, including sea urchins, fish, and other mesograzers (like decapods, gastropods, or isopods), is a crucial driver of the status of these systems and represents a potential threat to disturbed macroalgal forests (i.e., under the influence of overfishing, warming, invasive species; Monserrat et al., 2023; Sala et al., 1998, Sala et al., 2011). Thus, overgrazing has been reported as a cause for the degradation or collapse of marine forests, resulting in the prevalence of turf and barren state systems (Ling et al., 2009, 2015; Vergés et al., 2014). Three species of sea urchin, *Paracentrotus lividus* (Lamarck 1816), *Arbacia lixula* (Linnaeus 1758), and *Diadema africanum* (Rodríguez, Hernández, Clemente & Coppard 2013), are the most common benthic macroherbivores of sublittoral rocky bottoms in the Macaronesian ecoregion (NE Atlantic Ocean; Alves et al., 2001, 2003; Bernal‐Ibáñez, Gestoso, et al., 2021). In this region, numerous studies have explored the role of *Diadema africanum* over the rocky bottoms and how overgrazing has impacted subtidal macroalgal communities (Alves et al., 2003; Friedlander et al., 2017; Hernández et al., 2006). However, the potential influence of intertidal species, such as *P. lividus*, and their interaction with early‐life stages of *Cystoseira* sl. species has not been assessed in the region.

Considering the widespread deforestation of Mediterranean and Macaronesian macroalgal forests (Bernal‐Ibáñez, Cacabelos, et al., 2021; Bernal‐Ibáñez, Gestoso, et al., 2021; Hernández et al., 2005; Sangil et al., 2018; Thibaut et al., 2005) and the loss of important ecosystem services, significant efforts have recently been made to promote their protection and restoration (Cebrian et al., 2021; Smith et al., 2023; Verdura et al., 2018). Currently, techniques based on recruit enhancement (by obtaining new recruits from both ex situ and in situ procedures) are increasingly being used to restore marine forests (Falace et al., 2018; Verdura et al., 2018). However, herbivory pressure remains a significant challenge when planning marine forest restoration actions, as it is one of the main causes of failure (Monserrat et al., 2023). Although experiments involving recruits and successful restoration activities have been developed in the Mediterranean Sea (Galobart et al., 2023; Gran García et al., 2022; Medrano et al., 2020; Verdura et al., 2018), assessing the performance of crucial early‐life stages under different scenarios, this is not the case for Macaronesia. In this region, efforts have focused on understanding the dynamics surrounding *Cystoseira* s.l. habitats (Alves et al., 2001; Sangil et al., 2018) or the ecophysiology of adults under different pressures (Bernal‐Ibáñez et al., 2022).

This study aimed to investigate the complex dynamics influencing the success of early‐life stages in *Cystoseira foeniculacea* by (1) investigating the site‐specific nature of recruit success considering potential differences driven by factors such as temperature, herbivory, and canopy presence; (2) evaluating the role of herbivory, particularly by sea urchins, on recruit density; (3) identifying a potential thermal threshold for *C. foeniculacea* recruits; and (4) identifying the best position for recruits settlement from under the canopy, in the edge of the canopy, or out of the canopy.

The hypotheses for the experiment were: (1) Recruits survival is negatively influenced by higher temperatures, herbivorous, and turf presence; (2) recruits excluded from herbivory have higher survival ratios; (3) elevated temperatures inhibit the survival and development of recruits; and (4) adult individuals may inhibit recruit development by competition for resources.

## MATERIALS AND METHODS

### Study site and ex situ recruitment

This study was conducted on the island of Madeira (32.7° N, 17° W). The coastline in Madeira is dominated by cliffs and boulders, except for some rocky platforms and tidal pools. The waters are oligotrophic, and sea‐surface temperature (SST) typically ranges between 17.0 and 23.5°C but increases annually (Bernal‐Ibáñez, Cacabelos, et al., 2021). This effect could be exacerbated within enclosed rock pool environments (Bernal‐Ibáñez pers. obs.).

**FIGURE 1:**
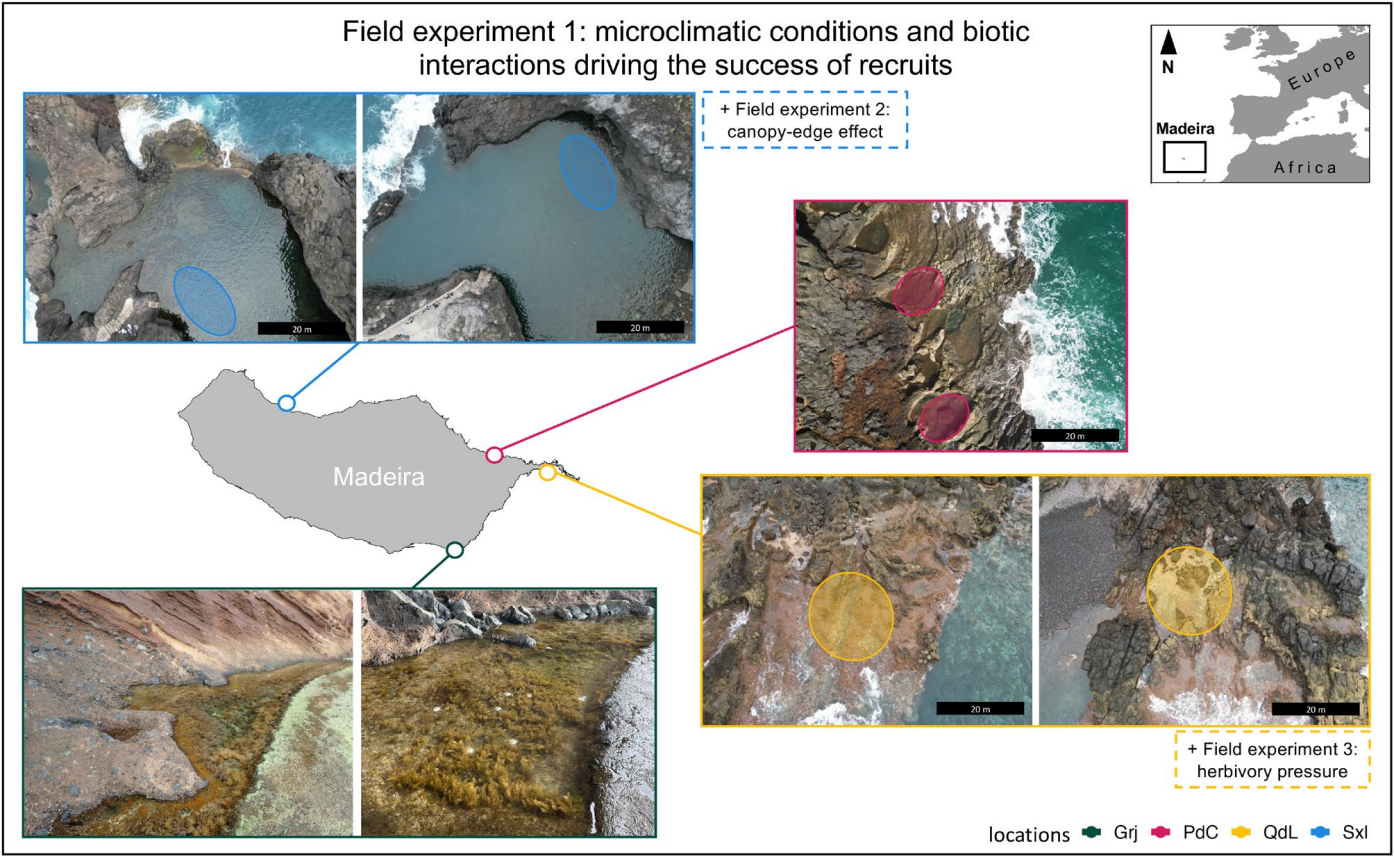
Map and pictures showing the different locations and rockpools included in this study: Clockwise from top Seixal (Sxl; donor population), Porto da Cruz (PdC), Quinta do Lorde (QdL), and Garajau (Grj). Circles in each picture represent where tiles were deployed within each rockpool. [Color figure can be viewed at wileyonlinelibrary.com]

Our target species was *Cystoseira foeniculacea*, a hermaphroditic species (Dawson, 1941) typically located in intertidal and shallow subtidal habitats in the NE Atlantic Ocean (Blanfuné et al., 2022), but nowadays in Madeira, it is only observed in some intertidal rockpools (Bernal‐Ibáñez, Gestoso, et al., 2021). The natural dispersal rate of *Cystoseira* s.l. species is limited (Mangialajo et al., 2012). Previous studies have shown that recruits settle between 0 and 2 m from adults (Gianni et al., 2013; Verdura et al., 2018). We harvested mature conceptacles by collecting around a total of 200 g fresh‐weight apical branches of *C. foeniculacea* containing fertile receptacles from at least 36 adults from a donor population located in a rockpool of Seixal (32.83° N, 17.11° W; Figure 1). We limited the collection of conceptacles to a single population due to the good status of adults in this rockpool and the low presence of this species around Madeira. Those apical branches were transported to laboratory facilities under cold and dark conditions. Once in the laboratory, they were carefully cleaned of any epibionts. The apical branches were stored at 4°C and in dark conditions before placing them in the culture tanks overnight (Verdura et al., 2018). The apical branches were placed over a mesh floating on the surfaces of ten 10‐L closed buckets in the MARE‐Madeira mesocosm system the day after. In the bottom of each tank, we placed twenty 3 × 3 cm^2^ natural rock tiles, previously brushed and cleaned with distilled water, as a substrate for the settlement of the zygotes (Verdura et al., 2018). Apical fertile branches were kept for 5 days on the surface of the tanks for the release of the zygotes. Once the recruits were observed on the tiles, we provided a continuous aeration source in each tank. The recruits were kept for 50 days in the mesocosm facilities, in an open system with filtered seawater (10 μm mesh) at a rate of 20 mL · min^−1^, resulting in a complete water turnover every hour at a constant natural temperature for the time of the year (19°C, January 2023). Two water pumps (Aqua Medic EcoDrift 4.1) were placed inside each water bath to ensure temperature homogeneity. We set four LED bars (eco + LED bar REEF 11000 K, 80 cm) on top of each water bath providing to each bucket an artificial photoperiod of 12:12 h (light:dark) with a quantum flux density (PAR) of 55.20 ± 6.31 μmol photons · m^−2^ · s^−1^ (mean ± *SD*, *n* = 18).

Before transplantation for all the experiments described in the following sections, we assessed the length of the recruits by detaching some individuals and measuring them under a microscope equipped with a Leica MC170 HD camera and software LASV4.12. The length size was 6.12 ± 0.10 mm (mean ± *SE*, *n* = 16; Figure S1 in the Supporting Information).

### Field experiment 1: Assessing the success of recruits under natural conditions

We transplanted 48 tiles with recruits of *Cystoseira foeniculacea* in four locations around Madeiran coastline for the main experiment: Garajau (Grj), Porto da Cruz (PdC), Quinta do Lorde (QdL), Seixal (Sxl; Figure 1). We chose these locations based on the following two criteria: (1) They are areas less exposed to wave action, facilitating the initial deployment of tiles and their subsequent monitoring and (2) the presence–absence of *C. foeniculacea* populations. Seixal and Grj present rockpools with *Cystoseira* communities, whereas in PdC and QdL, rockpools are dominated by turf and sea urchins. In each location, two rockpools were chosen, and six tiles were fixed in each pool with epoxy putty (Cebrian et al., 2021; Verdura et al., 2018).

Recruit density on experimental tiles and the surrounding benthic community was assessed monthly in each rockpool. For the community sampling, we took 12 random photoquadrats, which were later analyzed to assess the cover of canopy species, turf species, barren areas, and the number of sea urchins (see Results). We took a monthly picture of each tile (time 0, time 1, and time 2) with an Olympus TG‐6 camera in macro mode. The density of recruits was assessed by counting the individuals in each tile picture using the Multipoint tool from ImageJ software (Schneider et al., 2012). Furthermore, as close as possible to the tiles, we deployed three HOBO Data Loggers (UA‐002‐64) in each rockpool for temperature and light data.

### Field experiment 2: Canopy‐edge effect

**FIGURE 2:**
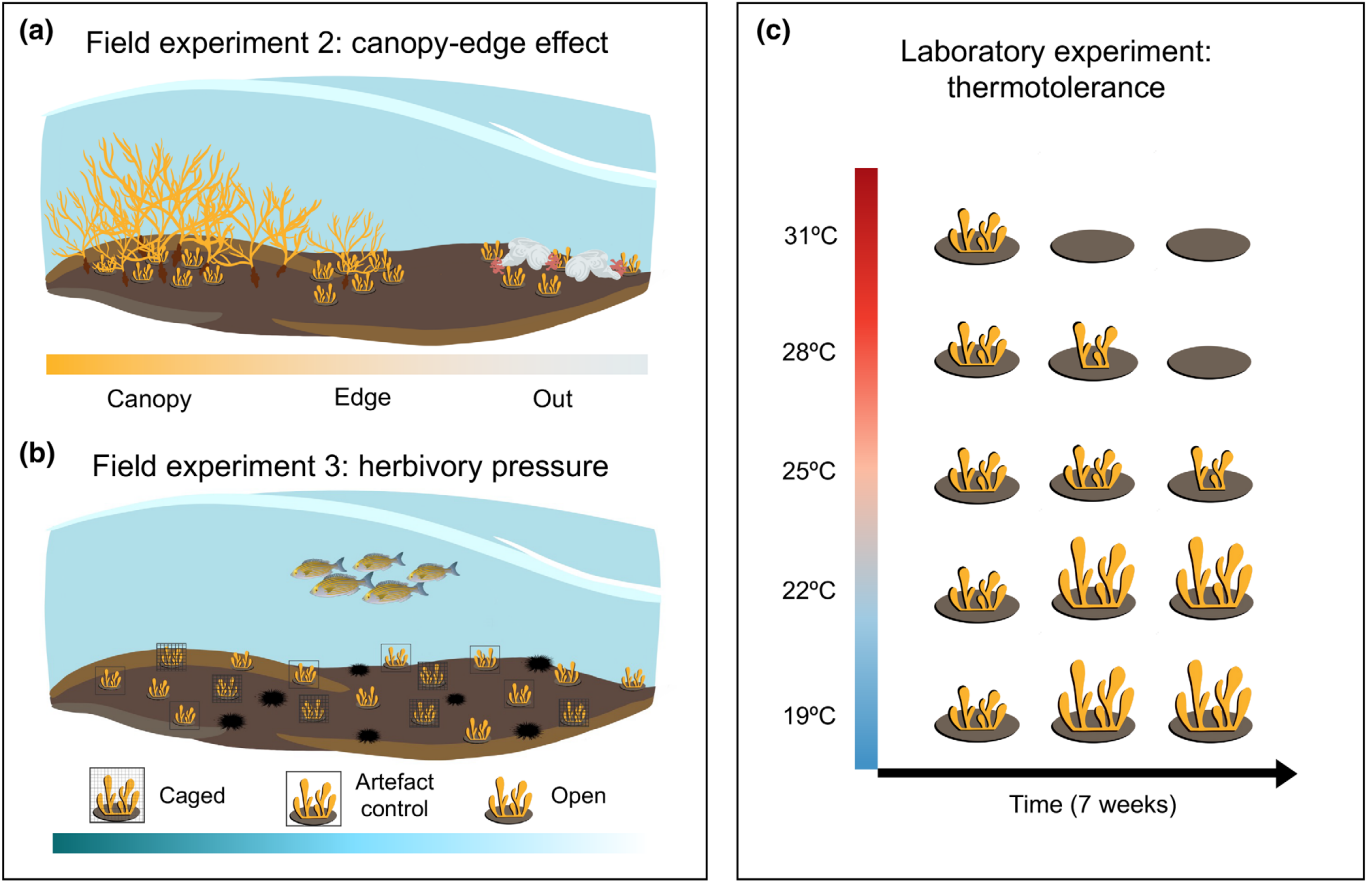
(a) Field experiment 2: Assessing the potential effect of the position over the success of recruits (inner part of the canopy, canopy edge, or out of the patch). (b) Field experiment 3: Exclusion of herbivory by sea urchins and fish through caging. (c) Laboratory experiment: Thermotolerance of recruits with temperatures ranging from 19 to 31°C over 7 weeks. [Color figure can be viewed at wileyonlinelibrary.com]

In one of the rockpools in Sxl, a *Cystoseira foeniculacea* dominant habitat, 12 extra tiles with recruits were fixed (total *n* = 18) to evaluate the effect of canopy cover on the success of the recruits in these rockpools (Figure 2a). For that, tiles were distributed in three different zones: the inner part of the canopy, the edge of the canopy, and out of the canopy (*n* = 6; Figure 2a). The density of *C. foeniculacea* recruits (number of recruits ∙ cm^−2^) was evaluated as the response variable and was monitored by taking a picture of each tile at time 0 (when fixed in the field) and after 1 month. Then, the density of recruits was assessed by counting the individuals in each tile picture with ImageJ software (Schneider et al., 2012) as mentioned in the previous sections.

### Field experiment 3: Herbivory pressure

Furthermore, we fixed an additional 18 tiles in a turf and sea urchin‐dominated rockpool in QdL to assess the effect of herbivory on recruits (Figure 2b). Six fixed tiles were protected to avoid herbivory (mainly by sea urchins and fish), six were covered with an artifact control, and six were maintained totally open (Figure S2 in the Supporting Information). The density (number of recruits ∙ cm^−2^) of *Cystoseira foeniculacea* recruits was evaluated using the previously described method.

### Laboratory experiment: Thermotolerance

We conducted a laboratory experiment in MARE‐Madeira mesocosm facilities to evaluate the thermotolerance response of *Cystoseira foeniculacea* recruits over a period of 7 weeks. For this, 15 tiles with recruits were subjected to five different temperatures ranging from 19 to 31°C (*n* = 3 per temperature; Figure 2c). Each tile was placed in an isolated 10‐L bucket (Figure S3 in the Supporting Information).

For the experimental setup, five water bath tanks (350 L each, one for each temperature) were used to maintain a water bath at the selected temperature (19, 22, 25, 28, and 31°C). Each water bath tank hosted three 10‐L buckets, each containing a tile with similar densities of recruits (Figure S3). Each bucket received filtered seawater individually at a rate of 20 mL · min^−1^, resulting in a complete water turnover every hour. After placing all the tiles with recruits in the buckets, the temperature was raised at a rate of 1.5°C · h^−1^ from 19°C to the selected temperature for each treatment. Each water bath tank maintained a stable temperature through two heaters (Schego 600 W) connected to a controller (Profilux 4, 5.1‐D‐PAB power bars; GHL Advanced Technology) and a temperature sensor. The sensor controlled the temperature automatically in one of the buckets for each temperature treatment. Two water pumps (Aqua Medic EcoDrift 4.1) were placed inside each water bath to ensure temperature homogeneity. The light system was the same as described in Study site and ex situ recruitment.

The density of *Cystoseira foeniculacea* recruits was evaluated using the method describe above. The size of recruits was assessed by measuring three random detached individuals for each treatment under the microscope above mentioned. Density and size were measured once per week until density values reached zero at the maximum temperature treatment (31°C after 7 weeks).

### Data analysis

For field experiment 1, a general additive model (GAM) was used to test the fixed effect of “Sea urchins density” and random effects of “Temperature,” sampling “Time,” and “Rockpool” on the “Density of recruits.” These variables were chosen from a pool of biotic (“Sea urchins density,” “Canopy cover,” “Turf cover,” and “Barren cover”) and abiotic (“Temperature” and “Light”) variables assessed during the experiment period after applying a Pearson correlation test.

Data from field experiment 2 were analyzed by applying a two‐way analysis of variance (ANOVA) to check the effect of factors “Position in the canopy” and “Time” over the density of recruits. The analysis included the two factors as fixed and orthogonal categories “Position in the canopy” (three levels: “Canopy,” “Edge,” and “Out”) and Time (month; 2 levels: “0” and “1”).

For the results of field experiment 3, we applied a two‐way ANOVA to analyze the effect of factors “Caging” and “Time” on the density of recruits. The analysis included the two factors as fixed and orthogonal categories “Caging” (three Levels: “Caged,” “Artifact control,” and “Open”) and “Time” (days; three levels: “0,” “2,” and “7”). A Tukey's HSD post hoc test determined significant differences between factor levels and possible interactions. Mean values were expressed with the standard error of the mean (mean ± *SE*).

The results from the thermotolerance experiment were analyzed by applying a linear model to test the effect of the temperature on the density and size (mm) of recruits, with “Temperature” (5 levels: “19°C,” “22°C,” “25°C,” “28°C,” and “31°C”) and “Time” (week; eight levels: “0,” “1,” “2,” “3,” “4,” “5,” “6,” and “7”). Previously, the density was standardized as relative recruit density (RD), as the change in density at a given time (RD_t_) relative to the initial measures (initial density RD_ii_) as relative recruits density = (RD_t_ − RD_i_)/RD_i_.

All statistical analyses were conducted in R version 4.4.1 (R Core Team, 2024), and a 5% significance level was applied. For field experiments 2 and 3 and the thermotolerance experiment prior to analysis, the homogeneity of variances was tested through Levene's test. A Tukey's HSD post hoc test determined significant differences between factor levels and possible interactions. Mean values were expressed with the standard error of the mean (mean ± *SE*). Data were visualized using the package ggplot2 (Wickham, 2011).

## RESULTS

### Field experiment 1: Assessing the survival of recruits under different natural conditions

**FIGURE 3:**
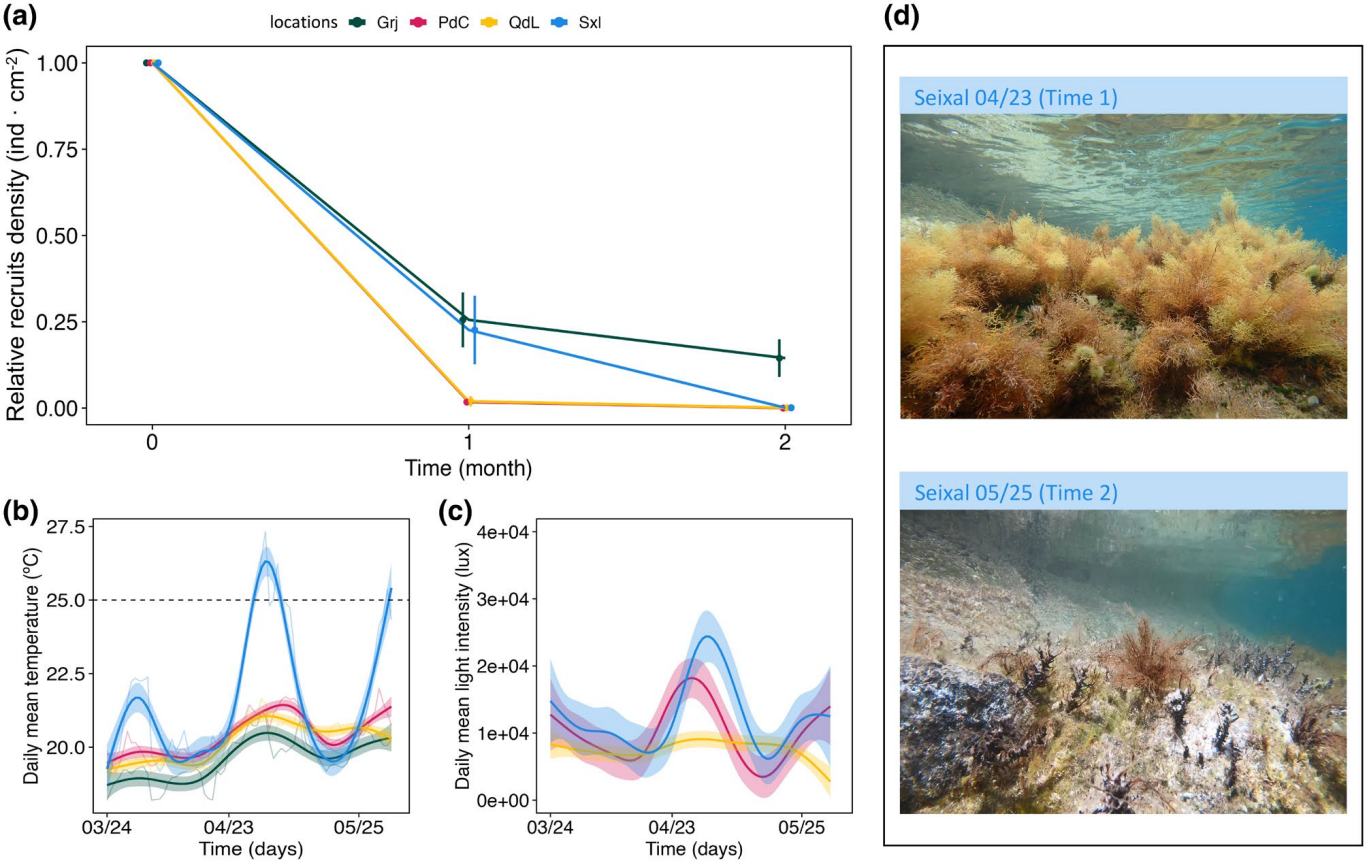
(a) Lineplot of the relative density of recruits (*n* = 6) for each location as a function of time (months 0, 1, and 2). Vertical bars represent standard errors. (b, c) Daily mean temperature and light intensity (Lux) were recorded in each location across time. The lines show the predicted values of generalized additive models (GAM) for each location and their confidence interval (95%). (d) Pictures of the *Cystoseira foeniculacea* population in Seixal's rockpools before (time 1) and after (time 2) the extreme temperature event of 11 consecutive days with a daily mean temperature >25°C. [Color figure can be viewed at wileyonlinelibrary.com]

In general, the density of recruits was negatively correlated with time, temperatures, and turf cover but positively correlated with canopy cover (Figure S4 in the Supporting Information). The temperature (as consecutive numbers of days with a mean >25°C) and time had an interactive effect on the density of recruits of *Cystoseira foeniculacea* (GAM, *F*_3,20_ = 4.95, *p* < 0.05; Table 1). Garajau and Sxl, where mature populations are located, presented similar densities of recruits after 1 month (Figure 3a). Further, in PdC and QdL, the turf‐barren‐dominated areas, the recruit densities were close to zero after 1 month. Between times 1 and 2, a marine heatwave was registered in the rockpools of Sxl, with 11 days of mean temperatures >25°C (Figure 3b) and a drop in the density values to zero after the event (Figure 3a). This affected the community in the pools, producing a shift phase from a canopy‐dominated habitat to the rise of turfs (Figure 3d; Figure S5a,b in the Supporting Information) Meanwhile, Grj, not affected by these extreme temperatures, still presented recruits (Figure 3a).

**TABLE 1:** ANOVA summary for the GAM fit to recruit density data (ind. · cm^−2^) and the effect of the factors included.

Source	*df*	Density of recruits (ind. · cm^−2^)	
		MS	*F* (*p*‐value)
Sea urchins density	1	0.26	4.95*
s(Temperature)	1	0	0
s(Time)	1	22.21	411.16**
s(Rockpool)	0.84	0.30	5.46*

*Note*: Sig. codes: **0.001 and *0.05.

Abbreviations: ANOVA, analysis of variance; GAM, generalized additive models.

### Field experiment 2: Canopy‐edge effect

**FIGURE 4:**
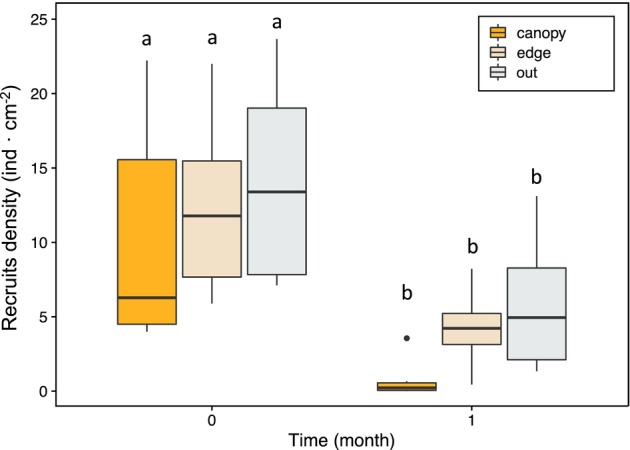
Boxplot showing the density of recruits of *Cystoseira foeniculacea* as a function of time (0 and 1) for the different “Position” treatments (canopy, edge, or out; *n* = 6) performed in Sxl. The bold horizontal lines indicate the median value (Q2), the box marks the interquartile distances (Q1 and Q3), and the whiskers mark the values less than Q3 + 1.5 * IQR but greater than Q1 − 1.5 * IQR. Different letters represent significant differences based on the Tukey's HSD post hoc test (*p* < 0.05). [Color figure can be viewed at wileyonlinelibrary.com]

Results showed a clear decrease in the density of recruits for all the treatments after 1 month. Density of recruits was significantly affected by time (month; ANOVA, *F*_2,15_ = 0.001, *p* < 0.001) but not by their position within the canopy (Table 2). However, maximum densities after 1 month were observed outside the canopy, with similar values in the edge and minimum inside the canopy, suggesting a negative gradient of survivorship from out to the inner part (Figure 4).

**TABLE 2:** Three‐way ANOVA summary showing the effect of “Position in the canopy” and “Time (month)” on recruits density (ind. · cm^−2^).

Source	*df*	Density of recruits (ind. · cm^−2^)	
		MS	*F* (*p*‐value)
Position	2	60.70	0.16
Time (month)	1	669.30	0.00*
Position × Time (month)	2	1.50	0.95
Residuals	30	30.6	

*Note*: Sig. codes: *0.001.

Abbreviation: ANOVA, analysis of variance.

### Field experiment 3: Herbivory pressure

**FIGURE 5:**
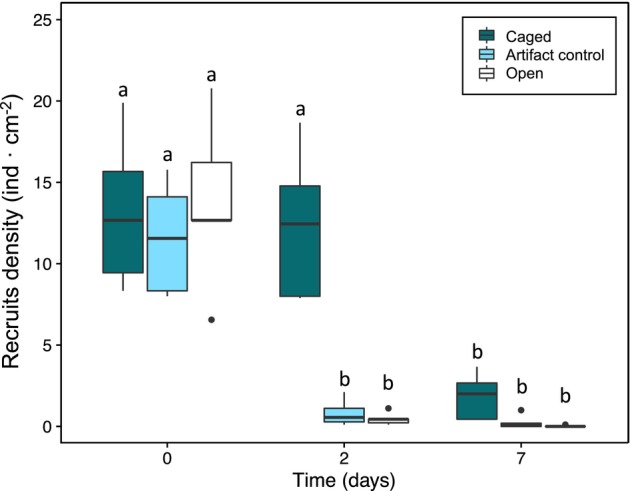
Boxplot showing the density of recruits of *Cystoseira foeniculacea* as a function of time (0, 2, and 7 days) for the different herbivory exclusion treatments (caged, artifact control, or open; *n* = 6) performed in QdL. The bold horizontal lines indicate the median value (Q2), the box marks the interquartile distances (Q1 and Q3), and the whiskers mark the values less than Q3 + 1.5 * IQR but greater than Q1 − 1.5 * IQR. Different letters represent significant differences based on the Tukey's HSD post hoc test (*p* < 0.05). [Color figure can be viewed at wileyonlinelibrary.com]

Density of recruits was significantly affected by the interactive effect of Caging × Time (days; ANOVA, *F*_2,15_ = 6.81, *p* < 0.001; Table 3). After 2 days, densities in artifact control and open treatments significantly decreased compared to caged individuals. After 7 days, density of recruits in caged tiles decreased but still presented higher values compared to artifact control and open (Figure 5).

**TABLE 3:** Three‐way ANOVA summary showing the effect of “Caging” and “Time (days)” on recruit density (ind. · cm^−2^).

Source	*df*	Density of recruits (ind. · cm^−2^)	
		MS	*F* (*p*‐value)
Caging	2	110.6	11.629*
Time (days)	2	581.6	61.17*
Caging × time (days)	4	64.7	6.81*
Residuals	36	9.5	

*Note*: Sig. codes: *0.001.

Abbreviation: ANOVA, analysis of variance.

### Laboratory experiment: Thermotolerance

**FIGURE 6:**
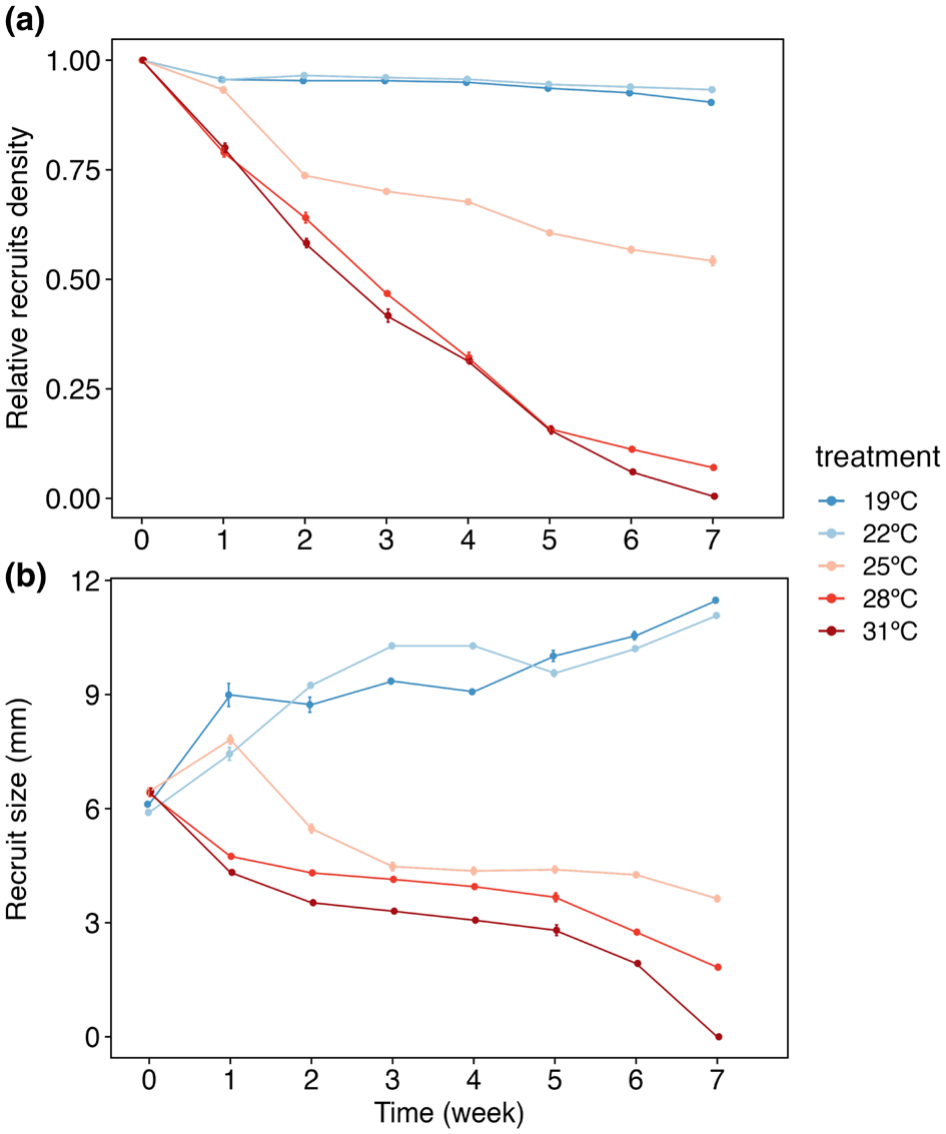
Relative density (a) and size (b) of recruits of *Cystoseira foeniculacea* as a function of temperature during the thermotolerance experiment (7 weeks; *n* = 6). Vertical bars represent standard errors. [Color figure can be viewed at wileyonlinelibrary.com]

Relative recruit density (RD) was significantly affected by the interactive effect of Temperature × Time (ANOVA, *F*_4,11_ = 178.98, *p* < 0.001; Table 4). After 7 weeks, the RDs at 19 and 22°C were very similar to those values at the beginning of the experiment (Figure 6a), with values showing over 90% survival. Minimum RD values were observed at 28 and 31°C after 7 weeks, reaching 0% for all the tiles at 31°C. Intermediate density values, around 54% survival, were reached for the tiles under 25°C (Figure 6a).

**TABLE 4:** ANOVA summary for the LM fit to relative recruit density data (ind. · cm^−2^) and the effect of the factors temperature and time (weeks).

Source	*df*	Relative density of recruits (ind. cm^−2^)	
		Sum^2^	*F* (*p*‐value)
Temperature	4	6.54	536.76*
Time (weeks)	1	3.32	1089.50*
Temperature × time (weeks)	4	2.18	178.98***
Residuals	110	0.34	

*Note*: Sig. codes: *0.001.

Abbreviations: ANOVA, analysis of variance; LM, linear model.

Recruit size was significantly affected by the interactive effect of Temperature × Time (ANOVA, *F*_4,11_ = 73.56, *p* < 0.01; Table 5). Maximum sizes were observed after 7 weeks at 19 and 22°C (Figure 6b). Conversely, the minimum values were observed at 28°C after 7 weeks, as we could not measure the size of the individuals at 31°C due to the low number of individuals and their poor health status. The size of the recruits under 25°C at the end of the experiment was 3.63 ± 0.08 mm (mean ± SE, *n* = 3; Figure 6b).

**TABLE 5:** ANOVA summary for the LM fit to recruit size data (mm) and the effect of the factors temperature and time (weeks).

Source	*df*	Size of recruits (mm)	
		Sum^2^	*F* (*p*‐value)
Temperature	4	818.98	287.01***
Time (weeks)	1	7.05	9.88*
Temperature × time (weeks)	4	209.90	73.56*
Residuals	110	78.47	

*Note*: Sig. codes: **0.001, *0.01.

Abbreviations: ANOVA, analysis of variance; LM, linear model.

## DISCUSSION

This study represents the evaluation of the performance of early‐life stages for a *Cystoseira* s.l. species in the Macaronesian region and examines the drivers shaping their survival under the current context of climate change. Similar to other habitat‐forming algae, *Cystoseira* s.l. species are threatened in most urbanized areas of the Mediterranean Sea and Macaronesia (Bennett et al., 2015; Bernal‐Ibáñez, Gestoso, et al., 2021; Mangialajo et al., 2008). Factors influencing the survival and population dynamics of *Cystoseira* species have been extensively studied, especially in adults (Capdevila et al., 2015; Irving et al., 2009; Pardi et al., 2000). However, the mechanisms shaping the recruitment and survival of early‐life stages remain incompletely understood, and further research is needed (Coelho et al., 2000). Periods with high temperatures occurring within the species' reproductive season can result in extensive mortality of zygotes. This threatens population survival and growth and produces a population bottleneck, as mortality of early‐life stages would prevent the natural replacement of adult individuals. The results of our main experiment have shown that even those environments dominated by mature canopies, where the conditions for the development of recruits are a priori optimal, can be exposed to discrete and extreme high‐temperature events causing the regression of the canopy and the total mortality of recruits. Nonetheless, this result may have been influenced by the fact that all the recruits were obtained from a single population, which may not provide a more general representation of the genetic diversity of this species. In any case, it is necessary to monitor microclimatic conditions, in this case in rockpools, where the abiotic dynamics of each rockpool may differ between locations or due to differences in rockpools size. This represents a limitation of our study, as the rock pools in Sxl are larger than those in the other locations, which may have induced differences between sites. Therefore, the survival of *Cystoseira* recruits in rockpools is highly site‐specific. Although the rockpools in Sxl were affected by a local marine heatwave, this did not occur in Grj, where the rockpools also present mature canopies dominated by *Cystoseira* sp. In Grj, recruits continued growing in consecutive months not included in this work (Bernal‐Ibáñez pers. obs.), and density remained stable. The initial decrease in the density of recruits in Grj but its subsequent stability could be explained by the competition density‐dependent effects: The density decreases as the size of recruits increases (Chapman, 1995). In the rest of the locations, where communities are dominated by turf and barren, the recruits did not survive after 1 month. This shows the high stability of the areas dominated by herbivores as a main driver of the maintenance of the status and the prevention of the recovery of canopies even when they are introduced artificially.

Previous studies have explored the effects of the presence of adults and the edge of the patches on the settlement and survival of *Cystoseira* sp. recruits (Capdevila et al., 2015; Piazzi et al., 2018). The impact of density‐dependent processes on the structure of algal populations has been controversial, with studies finding both positive and negative effects (Scrosati, 2005). Reproductive adults are essential for recruitment (Dudgeon et al., 2001), and they can provide protection to the recruits against environmental stressors, such as physical stress (Brawley & Johnson, 1991), or protection against grazing (Jenkins et al., 1999). However, it is considered that the recruitment of canopy‐forming species is disturbed by adult plants (Kendrick et al., 2019; Pardi et al., 2000). In fact, although adults may facilitate the early survival of newly settled propagules, the subsequent mortality of recruits seems to be greater under the canopy than in other habitats (Benedetti‐Cecchi & Cinelli, 1992). For example, the survival of recruits of *Cystoseira zosteroides* was shown to be strongly and negatively affected by adult patches (Capdevila et al., 2015). This is in accordance with our results; despite not finding significant differences in the canopy's effect on the survival of recruits, a negative pattern was observed in those recruits placed directly under the adults. We observed higher densities of recruits outside the influence of the canopy and minimum values under the influence of adults. It has been shown how mature canopies inhibit the survival of juvenile‐settled individuals by the abrasive action of adults (Vadas et al., 1992) and by competition for light and nutrients (Sjøtun et al., 2006). Light availability strongly influences the development of *Cystoseira* recruits, suggesting that this factor may play a key role in adult–recruit interactions (Irving et al., 2009) especially considering that the light requirements for recruits in Fucalean species might differ from adult plants and also broadly change during early ontogeny (Sanchez de Pedro et al., 2022). Patch edges often provide optimal conditions for species survival, but they are also the first areas susceptible to erosion and are more exposed to grazing (Bulleri & Benedetti‐Cecchi, 2006; Konar & Estes, 2003). The impact of the adult canopy on recruitment outcomes can vary along gradients of physical stress (Bennett et al., 2015). The reproductive strategy of short‐distance dispersal observed in *Cystoseira* species residing in shallow water may confer an adaptive advantage by facilitating the formation of monospecific stands near parent plants, particularly in habitats primarily influenced by physical factors (Mangialajo et al., 2008). We hypothesize that *Cystoseira* forests may grow toward patch edges, where recruits may find higher resource availability and less competition with adults than within the patch, where light and nutrients may be limited. However, the abrasive action of adult individuals and allelopathic effects may inhibit the development of smaller individuals (Benedetti‐Cecchi & Cinelli, 1992).

The herbivory exclusion experiment showed the rapid response of *Cystoseira foeniculacea* recruits to protection against grazing. The density of recruits changed in response to herbivory protection in just 2 days, indicating significant herbivory pressure affecting the recruitment success in areas dominated by sea urchins and other mesograzers. This highlights the importance of herbivory control in the early‐life stages for increasing restoration success. Furthermore, the short‐term effects observed in this study align with the patterns shown in a similar study recently conducted in the Mediterranean Sea (Monserrat et al., 2023). The herbivory pressure can vary depending on the characteristics of the experimental locations, such as physical environmental conditions and the structure of the benthic communities (Medrano et al., 2020). This study excluded sea urchins and fish, such as *Sarpa salpa* (Linnaeus, 1758), known to be efficient grazers on adults (Gianni et al., 2018). However, mesograzers were not excluded, and their role as grazers on recruits from other *Cystoseira* species has been recently assessed (Monserrat et al., 2023). This could explain the lower density of recruits in the caged tiles after 7 days, considering that mesograzers' herbivorous activity is slower than that of sea urchins or fish.

The mesocosm experiment conducted in this study identified 25°C as a thermal threshold for the development and survival of *Cystoseira foeniculacea* recruits. Few studies have specifically assessed the potential impacts of warming on early‐life stages of *Cystoseira* s.l. species. Previous research has examined the influence of elevated temperatures on the settlement and survival of recruits for these species (Capdevila et al., 2016; Verdura et al., 2021). Notably, a critical threshold of 24°C was identified for *Ericaria zosteroides*, a species residing in the deep‐sea environments in the Mediterranean Sea (Capdevila et al., 2018). For *Ericaria crinita*, a species from shallower water in the Mediterranean Sea, the thermotolerance threshold was determined to be 28°C (Verdura et al., 2021). The results of this study highlight that the thermotolerance of recruits from a Macaronesian population of *C. foeniculacea* is lower compared to other intertidal species in the Mediterranean Sea, making them more vulnerable to rising temperatures. This vulnerability could be associated with the observed regression of *C. foeniculacea* populations in the region in recent decades (Bernal‐Ibáñez, Gestoso, et al., 2021), a trend directly linked to the rise in temperatures observed in Macaronesia as well as to the increased frequency and intensity of marine heatwaves (Bernal‐Ibáñez et al., 2022; Bernal‐Ibáñez, Cacabelos, et al., 2021).

The observed short‐term effects on recruit density could have several long‐term implications, such as population decline (as reduced recruit density could lead to a decline in the overall population of *Cystoseira foeniculacea*), biodiversity loss (as *C. foeniculacea* provides habitat and shelter for various marine species), ecosystem function disruption (as canopy‐forming algae play a crucial role in coastal ecosystems, including in nutrient cycling and in providing food for herbivores), increased vulnerability of the population (as the population becomes more vulnerable to other stressors, such as climate change, pollution, and invasive species, potentially leading to further declines) or shifts in community structure.

This work has demonstrated the crucial role of developing and establishing continuous monitoring efforts in areas targeted for restoration, aiming to comprehend the dynamics of both abiotic and biotic factors at each site. Moreover, there is a pressing need for comprehensive studies that assess multiple impacts on the early‐life stages of *Cystoseira* sp. Although we combined field and mesocosm experiments to explore the effects of temperature, herbivory, and canopy presence on the development of early‐life stages of the brown seaweed, it is widely acknowledged that understanding the response of the marine environment to multiple stressful factors in combination is crucial for ecosystem‐based management. This is due to the complexity of the interactions among the factors and the ecosystem components, resulting in synergistic, antagonistic, or additive effects (Gissi et al., 2021). The low survival rates of recruits in natural environments show the necessity of unraveling interactions with other potential limiting factors and impacts. For instance, the adverse effects of warming on recruitment may be exacerbated by additional stressors, such as herbicides and pollutants, as previous research has shown these factors can affect the early developmental stages of *Cystoseira* s.l detrimentally (de Caralt et al., 2020). This present work, the first to evaluate the potential survival of *Cystoseira* sp. recruits in Macaronesia, lays the foundation for future regional‐scale studies, providing essential knowledge for the success of marine forest restoration policies.

## CONCLUSIONS

This study investigated the complex dynamics that influence the survival of the early‐life stages of *Cystoseira foeniculacea* in the Macaronesian region. Future work could consider the combined impacts of multiple stressors on crucial stages of habitat‐forming species development. We consider it necessary to replicate this type of study with other *Cystoseira* s.l. species to understand the complexity of the response in all the species. We emphasize the need for monitoring intertidal habitats, which are typically more exposed and vulnerable to rising temperatures in the current context of climate change. This will generate the necessary knowledge to develop successful restoration strategies, provided that climate change mitigation scenarios are met.

## AUTHOR CONTRIBUTIONS

**Alejandro Bernal‐Ibáñez:** Conceptualization (lead); data curation (lead); formal analysis (lead); investigation (lead); methodology (lead); visualization (lead); writing – original draft (lead); writing – review and editing (equal). **Eva Cacabelos:** Conceptualization (supporting); formal analysis (supporting); investigation (supporting); methodology (supporting); writing – original draft (supporting); writing – review and editing (equal). **Raul Triay‐Portella:** Conceptualization (supporting); investigation (supporting); methodology (supporting); writing – review and editing (supporting). **Patrício Ramalhosa:** Methodology (supporting); writing – review and editing (supporting). **Ignacio Gestoso:** Conceptualization (supporting); formal analysis (supporting); investigation (supporting); methodology (supporting); visualization (supporting); writing – original draft (supporting); writing – review and editing (equal).

## Supporting information


**Figure S1.** Recruits of *Cystoseira foeniculacea* from the ex situ recruitment and cultivation across time.

**Figure S2.** Herbivory exclusion experiment developed in a turf and sea urchins‐dominated rockpool. Different pictures show each treatment of the factor “Caging.”

**Figure S3.** (a) Laboratory facilities showing the mesocosm tanks. (b) Water bath treatment tank hosting each bucket with a tile. (c) Picture of a tile where recruits settled.

**Figure S4.** Corrplot showing the correlation between recruit density and biotic and abiotic variables assessed during the main experiment. Correlation values are only represented for significant correlations. Variables are time (month), norm_urchin_m2: normalized sea urchins density (ind. · m^−2^), norm: t cover: normalized turf cover, temp_dummy: temperature as a dummy variable (0 or 1 if values were above threshold 25°C), reldens_1: relative recruit density, station_num: rockpool, and norm_c_cover: normalized canopy cover.

**Figure S5.** Canopy cover (a), turf cover (b), barren cover (c), and sea urchin density (d) across time in the four stations. In (a), the red line is underneath the yellow line in the figure, so it is not visible except at the points.

## Data Availability

The data that support the findings of this study are available upon reasonable request from the corresponding author.
