# Supplementary figures and images for: Assessing climatic conditions and biotic interactions shaping the success of Cystoseira foeniculacea early‐life stages
Source: J Phycol. 2024 Oct 23;60(6):1485–97. doi: 10.1111/jpy.13516 (PMC11670282; doi:10.1111/jpy.13516)

1 day

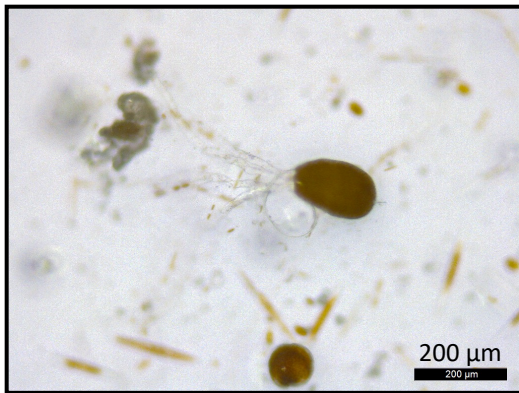

5 days

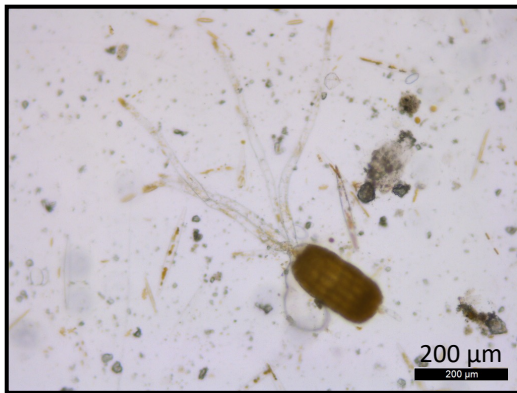

20 days

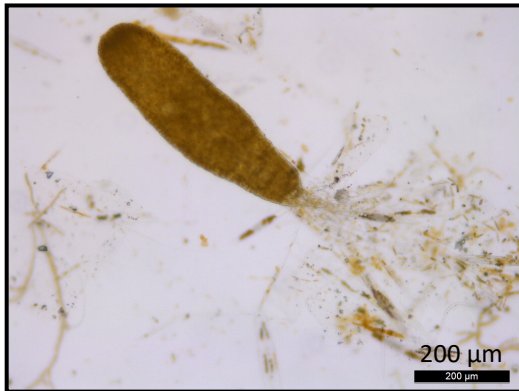

45 days

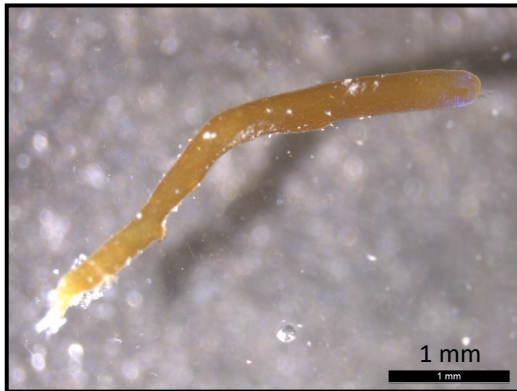

Supplement: Supplementary file 1 — Figure S1. Recruits of Cystoseira foeniculacea from the ex situ recruitment and cultivation across time. [file JPY-60-1485-s004.pdf]

# Field experiment 3: herbivory pressure on recruits

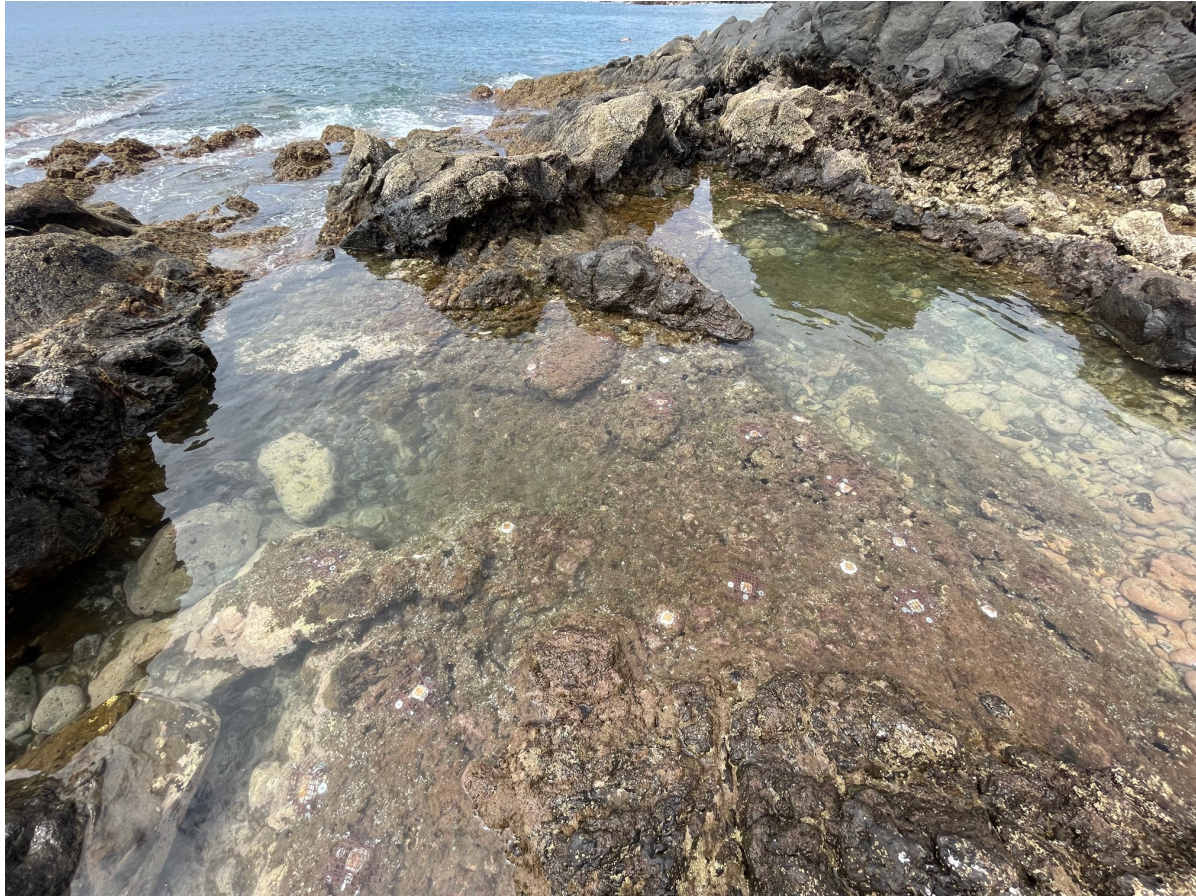

CAGED

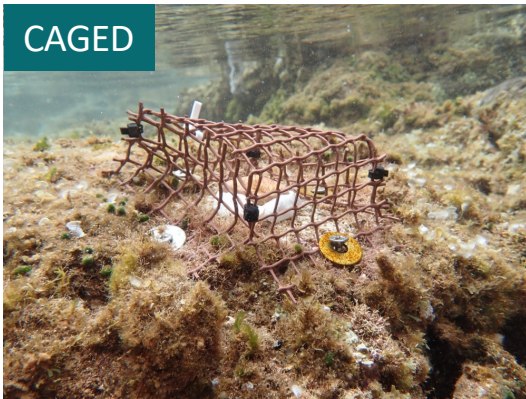

ARTEFACT CONTROL

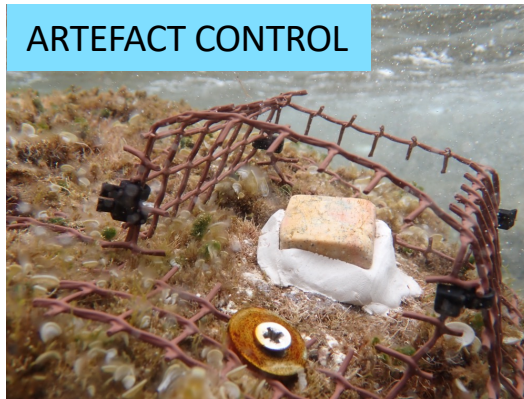

CONTROL

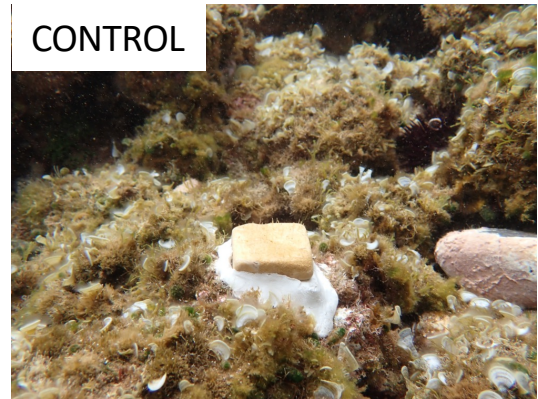

Supplement: Supplementary file 2 — Figure S2. Herbivory exclusion experiment developed in a turf and sea urchins‐dominated rockpool. Different pictures show each treatment of the factor “Caging.” [file JPY-60-1485-s003.pdf]

# Laboratory experiment: recruit's thermotolerance

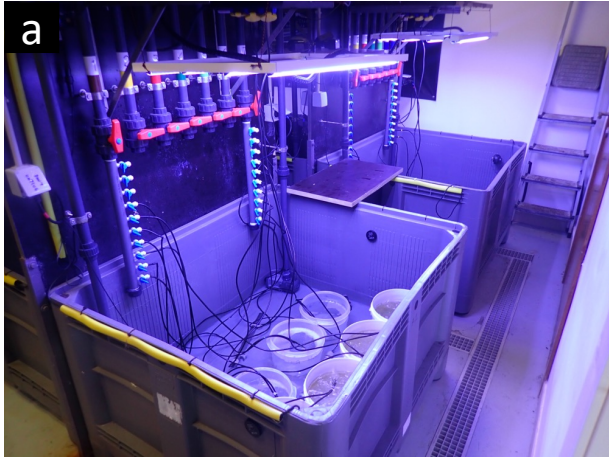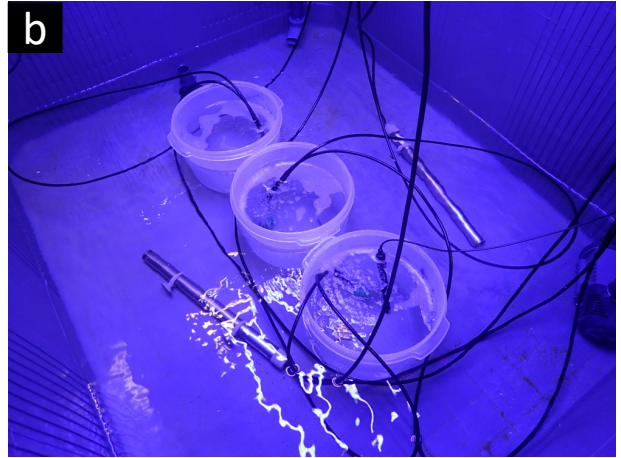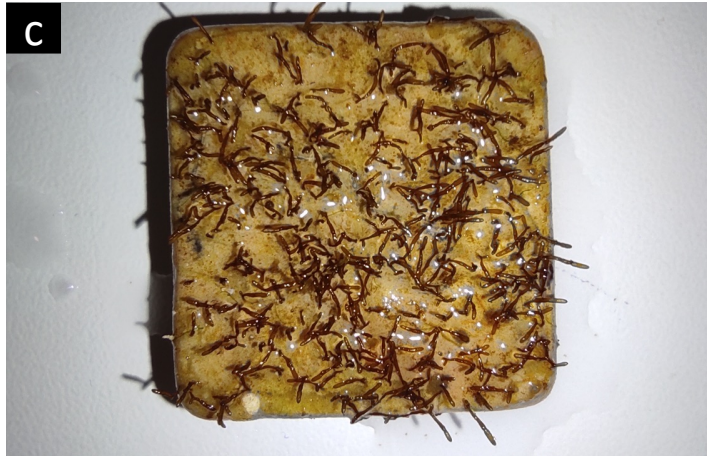

Supplement: Supplementary file 3 — Figure S3. (a) Laboratory facilities showing the mesocosm tanks. (b) Water bath treatment tank hosting each bucket with a tile. (c) Picture of a tile where recruits settled. [file JPY-60-1485-s002.pdf]

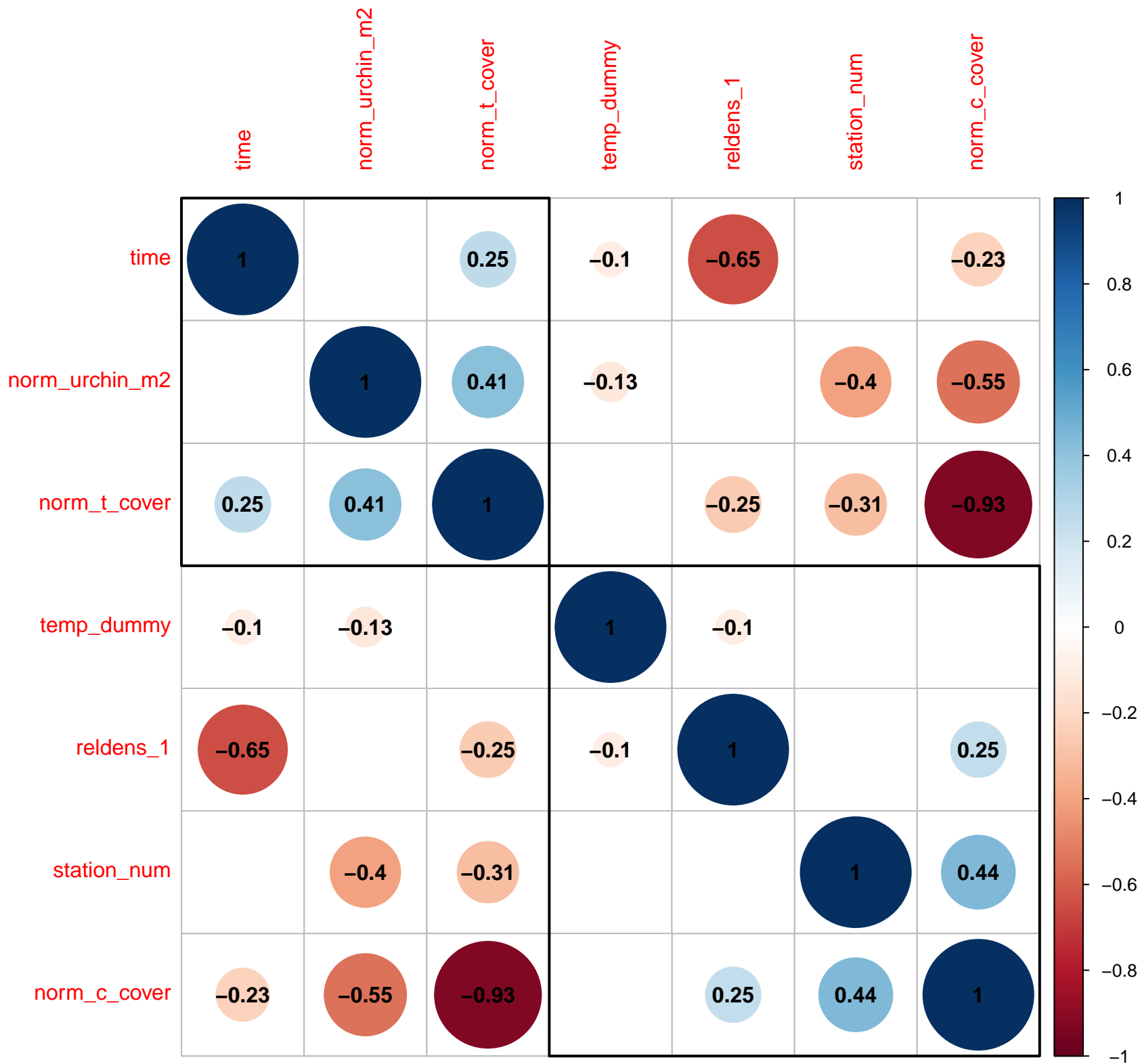

Supplement: Supplementary file 4 — Figure S4. Corrplot showing the correlation between recruit density and biotic and abiotic variables assessed during the main experiment. Correlation values are only represented for significant correlations. Variables are time (month), norm_urchin_m2: normalized sea urchins density (ind. · m−2), norm: t cover: normalized turf cover, temp_dummy: temperature as a dummy variable (0 or 1 if values were above threshold 25°C), reldens_1: relative recruit density, station_num: rockpool, and norm_c_cover: normalized canopy cover. [file JPY-60-1485-s005.pdf]

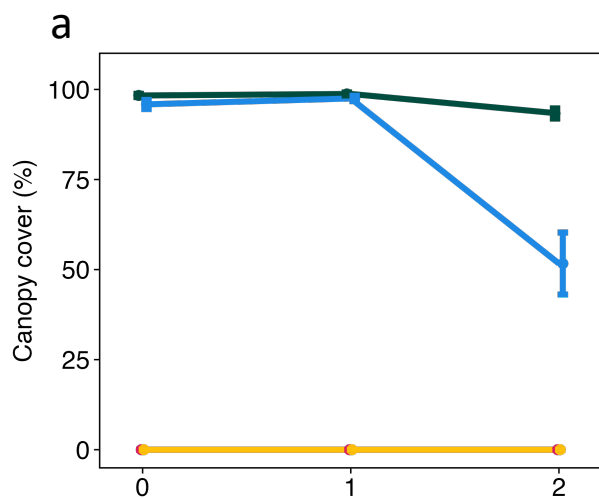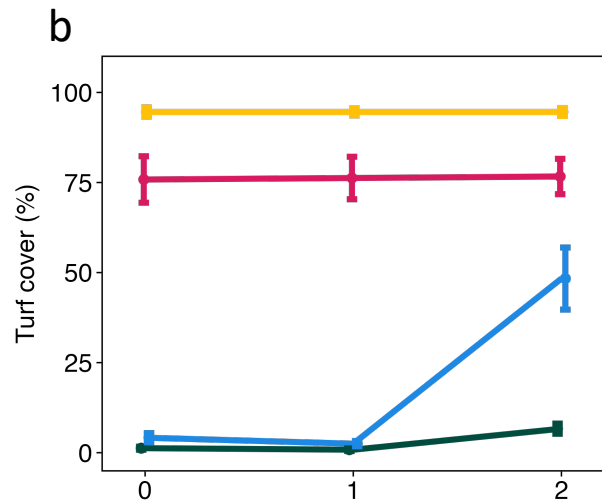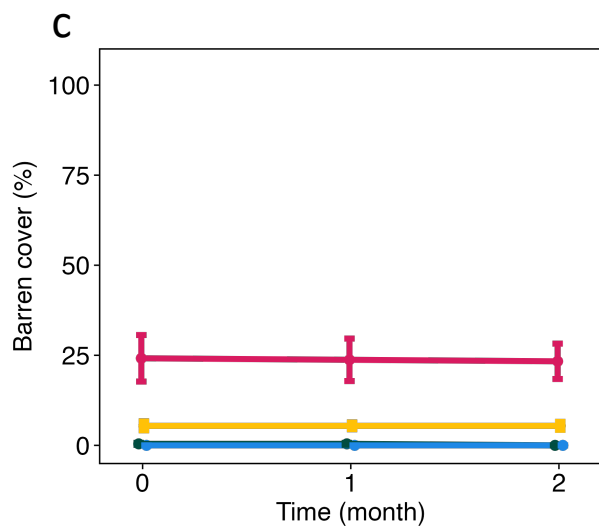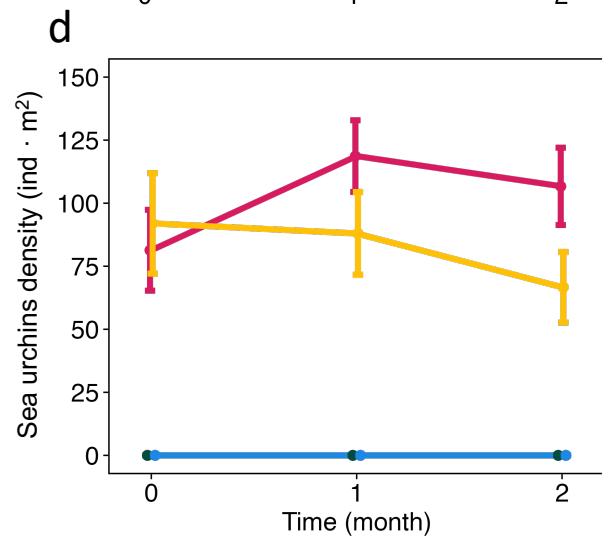

locations: Grj PdC QdL Sxl

Supplement: Supplementary file 5 — Figure S5. Canopy cover (a), turf cover (b), barren cover (c), and sea urchin density (d) across time in the four stations. In (a), the red line is underneath the yellow line in the figure, so it is not visible except at the points. [file JPY-60-1485-s001.pdf]
